# An 8.72 µW Low-Noise and Wide Bandwidth FEE Design for High-Throughput Pixel-Strip (PS) Sensors

**DOI:** 10.3390/s21051760

**Published:** 2021-03-04

**Authors:** Folla Kamdem Jérôme, Wembe Tafo Evariste, Essimbi Zobo Bernard, Maria Liz Crespo, Andres Cicuttin, Mamun Bin Ibne Reaz, Mohammad Arif Sobhan Bhuiyan

**Affiliations:** 1 Energy, Electrical and Electronics Systems, Department of Physics, University of Yaoundé I, P.O. Box 812, Yaoundé 222, Cameroon; jfolla_1@ictp.it (F.K.J.); bernard.essimbi@uy1.uninet.cm (E.Z.B.); 2 Multidisciplinary Laboratory (MLAB), International Centre for Theoretical Physics (ICTP), Via Beirut 31, 34100 Trieste, Italy; mcrespo@ictp.it (M.L.C.); cicuttin@ictp.it (A.C.); 3 Laboratory of Electronics and Automatics, Department of Physics, University of Douala, P.O. Box 24157, Douala 233, Cameroon; evaristewembe@univ-douala.com; 4 Electrical, Electronic and Systems Engineering, Universiti Kebangsaan Malaysia, Bangi 43600, Selangor, Malaysia; mamun@ukm.edu.my; 5 Electrical and Electronics Engineering, Xiamen University Malaysia, Bandar Sunsuria, Sepang 43900, Selangor, Malaysia

**Keywords:** ASIC, CMOS technology, compact muon solenoid, integrated front-end electronics, low-noise, low-power, radiation sensor

## Abstract

The front-end electronics (FEE) of the Compact Muon Solenoid (CMS) is needed very low power consumption and higher readout bandwidth to match the low power requirement of its Short Strip application-specific integrated circuits (ASIC) (SSA) and to handle a large number of pileup events in the High-Luminosity Large Hadron Collider (LHC). A low-noise, wide bandwidth, and ultra-low power FEE for the pixel-strip sensor of the CMS has been designed and simulated in a 0.35 µm Complementary Metal Oxide Semiconductor (CMOS) process. The design comprises a Charge Sensitive Amplifier (CSA) and a fast Capacitor-Resistor-Resistor-Capacitor (CR-RC) pulse shaper (PS). A compact structure of the CSA circuit has been analyzed and designed for high throughput purposes. Analytical calculations were performed to achieve at least 998 MHz gain bandwidth, and then overcome pileup issue in the High-Luminosity LHC. The spice simulations prove that the circuit can achieve 88 dB dc-gain while exhibiting up to 1 GHz gain-bandwidth product (GBP). The stability of the design was guaranteed with an 82-degree phase margin while 214 ns optimal shaping time was extracted for low-power purposes. The robustness of the design against radiations was performed and the amplitude resolution of the proposed front-end was controlled at 1.87% FWHM (full width half maximum). The circuit has been designed to handle up to 280 fC input charge pulses with 2 pF maximum sensor capacitance. In good agreement with the analytical calculations, simulations outcomes were validated by post-layout simulations results, which provided a baseline gain of 546.56 mV/MeV and 920.66 mV/MeV, respectively, for the CSA and the shaping module while the ENC (Equivalent Noise Charge) of the device was controlled at 37.6 e^−^ at 0 pF with a noise slope of 16.32 e^−^/pF. Moreover, the proposed circuit dissipates very low power which is only 8.72 µW from a 3.3 V supply and the compact layout occupied just 0.0205 mm^2^ die area.

## 1. Introduction

**Figure 1:**
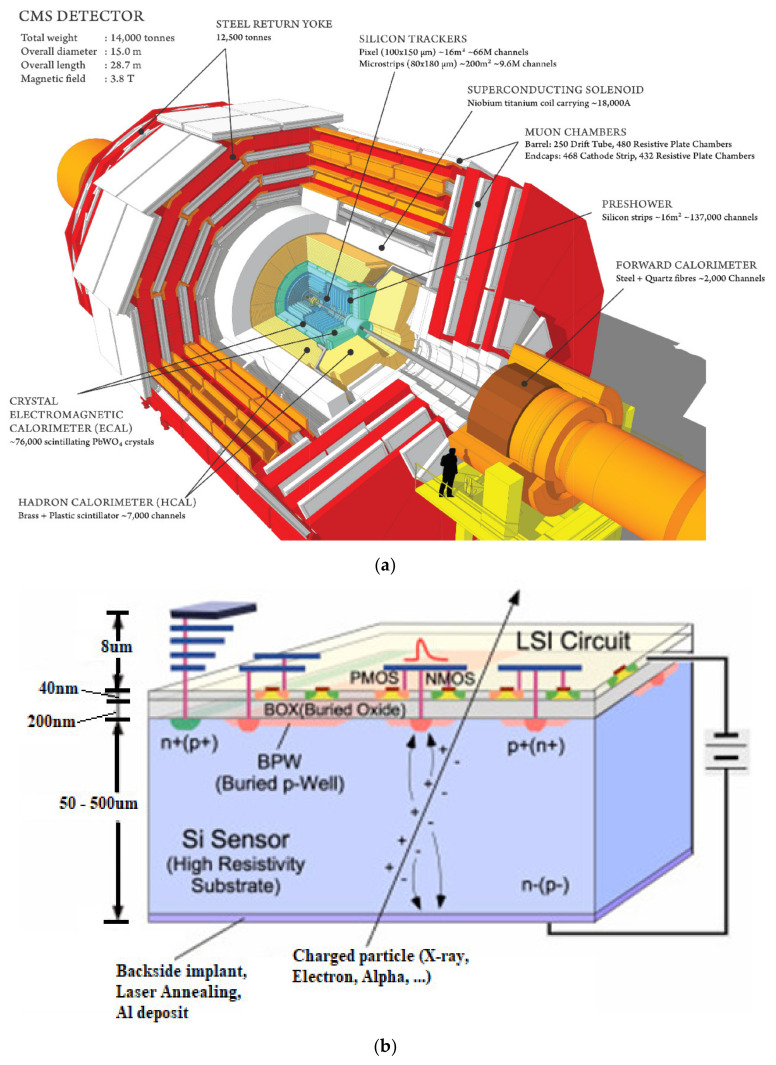
(**a**) The building blocks of the Compact Muon Solenoid (CMS) [5]. (**b**) Principle of operation of a silicon pixel-strip sensor [7].

The front-end readout system for modern High Energy Physics Experiments (HEPEs) is a mixed-signal circuit, which performs precise measurement of particle trajectories. It amplifies the output signal of the photon sensor. A data acquisition (DAQ) based Field Programmable Gate Array (FPGA)-based board then extracts all necessary data about the photons from the output signals of the readout electronics and utilizes that information to figure out a coincidence pair of photons to create a line of response (LOR) [1,2,3,4]. For instance, the Compact Muon Solenoid (CMS) illustrated in Figure 1a [5], is predicted to receive a substantial upgrade of the outer tracker sensor and its front-end readout electronics, needing higher granularity and readout bandwidth to absorb a big amount of pileup events in the High-Luminosity Large Hadron Collider (LHC) [2,5]. Therefore, the whole tracking system will be substituted with highly radiation-tolerant sensors which will be capable of handling higher readout bandwidths and particle flux rates [2,5].

To recognize particles having higher transverse momentum (>2 GeV/c) and to distinguish the front-end output with a given L1 trigger level, a double layer sensor module, which combines a pixel sensor with a strip one, was adopted. Consequently, two different readout application-specific integrated circuits (ASICs) were developed, namely the Short Strip ASIC (SSA) for the strip sensor and the Macro Pixel ASIC (MPA) for the pixelated sensor [2,5,6]. The operating principle of a pixel-strip sensor is illustrated in Figure 1b [7]. As ionization is produced on each strip, and the readout circuit should process the ionized particles; therefore, in order to handle higher particles flux, SSA is needed to be implemented within a Complementary Metal Oxide Semiconductor (CMOS) process and integrated into the sensor’s chip [2,5]; this will avoid loss of transmission between the high-speed interconnects and the readout ASIC chip [8,9,10,11].

Recent research on pixel-strip sensors reveals that those devices can transform gamma rays to charges operating at normal temperature, which exhibits a better potentiality for the detection of X-rays and γ-rays for possible nuclear instrumentation applications [6]. A typical thickness for Si-sensor is about 300 µm; the limiting irradiation energy, which would penetrate protons through the sensor, is about 6.2 MeV [5,7]. With moderate cooling by means of small Peltier cells, silicon drift detectors and Si-PIN sensors show particularly excellent spectroscopic performances and good detection efficiency below 15 keV [5,11,12]. In contrast to the spectroscopy amplifier, the major concern for a fast amplifier is the preservation of the charge collection process while keeping a wide bandwidth, which in turn optimizes the signal rise time [4,5,9,12]. The improvement of energy resolution leads to optimization of the charge collection process by designing the lowest possible rise time of the charge sensitive amplifier (CSA) compared to the peaking time of the shaping amplifier; this would prevent ballistic deficit, which involves loss of resolution. Therefore, the energy sensitivity of the readout module should be high enough to minimize the energy loss and guarantee a high rate collection process, which is characterized by its rise time (tr) and can be performed in less than 10 ns to guarantee high counting rate operations [4]. Moreover, for multi-channel readout electronics, the spatial resolution should be more than 2 µm [4,5,12].

**Figure 2:**
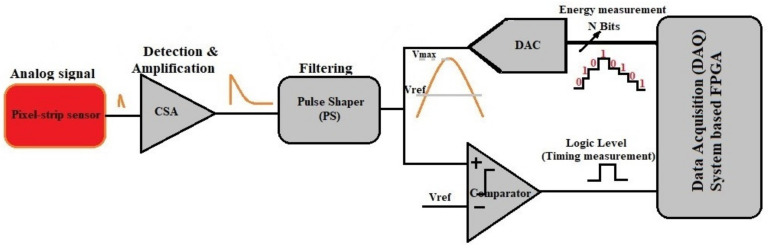
Pixel-strip sensor readout architecture for digital processing, the Charge Sensitive Amplifier (CSA) is used for extracting the charge at each strip and convert it into voltage.

A big amount of channels can be made feasible using large-scale integration to include the associated electronics on the same chip of the sensor. Silicon sensors offer a typical signal in the range of tens of thousands of electrons within a collection time of few nanoseconds that should be processed by a readout integrated circuit (ROIC). Signal processing starts with the integration of the input signal, a very small and fast current pulse, into a voltage step performed by CSA [8,9,10]. The CSA output swing is proportional to the total integrated charge, which is in time proportional to the energy released by the incident particles in the sensor. This energy must be measured with the highest accuracy and precision [2,3]. The input node voltage of the CSA increases (tends to increase) and the voltages with the opposite polarity are generated at the output terminal simultaneously. Hence, the output potential through the feedback loop forces the input potential of the CSA to become zero because of high open-loop gain as shown in Figure 2.

The input current pulse is integrated into the feedback capacitor and the corresponding output is a step voltage pulse [6,7,10,13,14]. This voltage is filtered and digitized by an Analog to Digital Converter (ADC) as shown in Figure 2. The resulting data are then coded into an appropriate format so that pixel address, time, amplitude or transverse momentum [5,12] can be extracted through an FPGA module for further processing [11,15].

It is well known that the input signals intercepted by CSA are generally very low in the range of few fC (~1fC) charges. For a given source, the generated preamplifier noise and the input impedance of the amplifier influence the front-end noise performance. The impact of radiations on the devices exacerbates the situation [9,11,12,16,17]. Therefore, the front-end input stage must ensure that optimum noise matching is achieved for the source impedance [11,12,17]. The design parameter of the input stage of CSA directly influences the noise matching. So, the equivalent input noise should be kept as minimum as possible for a given sensor capacitance. The main problem in the design of nuclear spectroscopy very large scale integration (VLSI) readout front end is the implementation of low-noise and low-power CSA. CMOS exhibits several advantages over other concurrent technologies (such as Bipolar, BiCMOS, etc.) and therefore, usually preferred to design application-specific integrated circuits (ASICs) [6,14,18,19]. A widely accepted front-end electronics (FEE) design approach is the use of an operational amplifier (Op-amp), with the R-C feedback network. However, this needs large sensor capacitance (about 15 pF), which compromises the stability of the design [6,14]. The stability, conditions are indicated by the phase margin (PM) and the gain-bandwidth product (GBP) within the Bode plot for the design of single-stage and two-stage amplifiers. However, the stability of multistage amplifiers requires advanced computations than single-or two-stage amplifiers; resulting from the existence of complex poles in high-order switch capabilities [6,20,21]. In addition, the desired performance requirements (GBP, PM) rely on the frequency compensation method and the value of the load capacitance C_L1_. For a complete validation of the front-end electronics with CMOS technology, the overall system specifications are needed [20,21,22]. In ref. [22], H. Wang et al., proposed readout electronics with CSA-based Polyvinylidene Fluoride (PVDF) transducers. The circuit works for low power dissipation and low frequency, but it was prone to low conversion gain, high feedback capacitance that occupies more die area. Moreover, due to several biasing points, that circuit was prone to more threshold variation and exhibited a higher dc-component, which worsen the output swing of the design [23,24]. In ref. [23], Haryong Song et al. proposed the Ripple Rejection Loop (RRL) techniques for mismatch reduction and offset cancellation in the input transistor stage. The technique works for low-frequency applications. However, the RRL circuit for X-rays and gamma rays spectroscopy could be implemented at the expense of some flicker noise and radiation damage [24,25], in high frequency. Moreover, due to power consumption requirements and hit transfer, the on-chip implementation of the RRL circuit is huge and is therefore not encouraged for spectroscopic purposes.

In recent years, radiation effects have become an important issue in semiconductor readout systems. Radiation hardened devices are constrained by the technology [7,9,26]. Scaling down technology leads to lowering the gate-oxide thickness, involving variations in threshold voltage (Vth) and inducing radiation damage. The reduction of threshold voltage shift (Vth variations) leads to minimizing the gate-oxide thickness (t_ox_) [9,26], then increasing the probability of quantum tunneling of electrons, which enables, therefore, most of the trapped holes caused by induced radiation to be recombined with electrons [26]. The low-threshold voltage (LVT) operation of subthreshold circuits applies lower electric fields across the gate-oxide [27]. This will reduce the rate of electron-hole separation and increase the probability of recombination. Therefore, this induces a lower trapped charge in the oxides and hence lower will be the radiation-induced threshold voltage shift and leakage current. Reducing variation of Vth helps the MOSFET device become more radiation-tolerant (more robust to radiation) [7,8,9,10,26]. A. Baschirotto et al. [20], designed a front-end using a single-ended amplifier as CSA. The circuit works at high frequency and very low voltage; however, the disadvantages of that circuit are high power consumption and high equivalent noise charge (ENC) which worsen the radiation-hardened behavior of the circuit [9,19,25,28,29]; furthermore, the circuit was prone to more parallel noise generated by the passive feedback resistor. The main problem in designing nuclear spectroscopy very large scale integration (VLSI) readout front ends is the execution of low-noise and low-power CSA, which guarantees high particles flux with the lowest pulse pile-up. Therefore, a good choice in the pulse shaping parameters is crucial for achieving good energy resolution and minimum pulse pile-up for high counting rates [11,30,31]. For high throughput experiments, short shaping time ($\tau_{s}$) reduces the pile-up effects and for an optimal design solution, the minimum $\tau_{s}$ limits the charge collection process and increases the energy resolution accordingly [4,12,25,26,27,28,29,30,31,32]. Therefore, it is necessary to propose an optimal front-end circuit to avoid unnecessary power dissipation and heat in closely packed pixel arrays first avoid. Secondly, the ENC should be optimized concerning sensor capacitance along with the shaping time and the input transistor width, for performing AC and transient analysis and finally, the core amplifier should guarantee a high loop gain, wide bandwidth, high stability and very low-power consumption [6].

This work describes the design and simulation of an ultra-low-power, low-noise and wide bandwidth FEE for high throughput pixel-strip sensors. The circuit consists of a three-stage single-ended CSA followed by a one-order Capacitor-Resistor-Resistor-Capacitor (CR-RC) pulse shaper (PS). The originality of this research results in the following statement; a modified CSA topology was designed for ultra-low-power and high counting rate solution. To compensate for the bandwidth limitation and achieve good stability along with preserving the pulse height degradation, an adjustable gain stage over a wide input dynamic was implemented and controlled by an external device. For this purpose, a common-source (CS) input design is adopted to segregate the input capacitance in order to avoid any bandwidth adjustment. Further, a Miller compensation with zero nulling resistors (MCNR) combined with external feedback was used to cancel out the second pole in the transfer function of the CSA open-loop gain thus, stabilizing the gain-bandwidth product of the circuit. A custom feedback network-based voltage-controlled N-type Metal Oxide Semiconductor (NMOS) resistor was also implemented to cancel out the parallel noise of the passive feedback resistor in the CSA module. A simple and optimal pulse sharper circuit was designed for achieving the highest possible signal-to-noise ratio (SNR) to allow a scale adjustment in energy resolution [11,12,32,33,34,35,36,37,38]. Further, rigorous transistor sizing/matching was performed to reduce the mismatch and achieve an ultra-low-power behavior of the circuit while assuring the radiation hardness behavior of the design [37,38,39,40,41,42]. The rest of the paper is organized as follows: Section 2 provides the design philosophy and materials. Analysis related to the CSA and shaper architectures are discussed, the design parameters are derived and implemented; therefore, the proposed front-end is validated and simulated. In Section 3, the achieved results are discussed. The paper is concluded in Section 4.

## 2. Design Philosophy and Materials

As illustrated in Figure 2 the global diagram of the front-end electronics is presented. The circuit consists of a CSA as a first stage followed by a differentiator and a one stage integrator as the shaping stage, which further amplifies the CSA output signal and optimizes the signal to noise ratio (SNR). This constitutes one channel of detection. The sensor, with a capacitance C_det_, produces current pulses that are integrated on the CSA feedback capacitor C_F_ [6,25,26,33]. To reduce the pile-up, it is necessary to use a short peaking time. The tradeoff of bandwidth, pulse rise time, peaking time and counting rate is necessary for the selection of the topology of the CSA core Op-Amp [4,25].

Several high gains with wide bandwidth CMOS Op-Amps have been developed and conveyed recently. Those topologies usually employ three to five gain-boosting stages to ensure high gain and mostly necessitate a number of compensation capacitors [34]. It is clear from the literature that the enhancement of the amplifier gain is achieved because of adopting positive feedback, which in turn produces a compensating negative conductance [29,35]. However, in most of those structures, the positive feedback generates a negative resistance at the output node, which produces high DC gain by compensating some of the positive resistance at the output [30,31]. 

The self-cascode structure also known as composite cascode structure is sometimes used to control the gain of CMOS Op-Amp, since they are built by cascading common source with a common gate; the structure offers a larger effective channel length and a larger effective output resistance [32]. However, at higher frequencies, the output capacitor starts shorting out, providing a low impedance path to the small-signal current and thus there is a decrease in gain. Combining this with the high DC-gain produced by the positive feedback structure will exacerbate the situation and introduce a poor gain measurement at high frequencies [34].

### 2.1. Design of the CSA Core Amplifier Circuit

**Figure 3:**
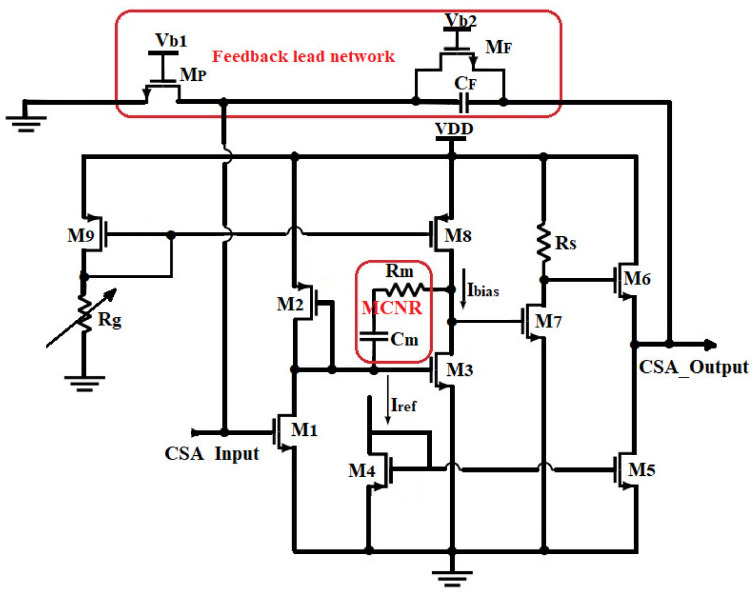
Schematic of the structure of the proposed CSA.

For high-speed applications, the GBW of the CSA must be made maximized [4,25]. To overcome the bandwidth limitation and improve the amplitude resolution for excellent particle identification ability, the GBW of the preamplifier is extended to achieve an output rise time of about a few ns as a response to impulsive charge [4]. This requires therefore high input transistor transconductance (gm) [6,28,34]. However, increasing the gate transconductance of the input transistor for a given drain current deals with increasing the device channel width and total gate capacitance, which worsens the electric noise. Therefore, optimizing the sizes of the MOSFETs would lead to a more radiation tolerant circuit [9,10]. Most of the shortcomings of the previous section can be eliminated by custom transistor sizing during the design process [28,30,31,32] along with implementing an internal compensation. In the former case, the compensation network is fabricated on the chip, and usually, no external access to the compensation network is provided [37]. A custom compensation technique in which the CSA GBW is adjusted by an external device is proposed. The proposed CSA has been designed in 0.35 µm technology from the TSMC process. The input transistor aspect ratio Width/Length (W/L) was suitably designed for low-noise and high gain purposes [11,12]. Moreover, an on-chip gain adjustable stage was implemented to extend the bandwidth of the core amplifier. An external resistor through a bias current controls this adjustable gain stage. A custom feedback network was adapted to perform the initial conversion of small current pulses into voltage step pulses. Table 1 presents the design specifications of a CSA circuit for typical Silicon-PIN sensor applications. To increase the gain of the CSA, we studied a three-stage configuration for the design. The single-ended configuration of the circuit exhibited in Figure 3, is more appropriate than the differential one for the reduction of power consumption. The choice of the N-channel input transistor relies on the lower thermal noise compared to the P-type at high frequency [9,18], since the 1/f noise is negligible in the frequency region above 10 kHz [6,38,39]. In addition, N-channel MOS, gives a lower series white noise with respect to the P-channel counterpart, because of its higher transconductance [6,27,38] at the same drain current compared to the PMOS device. The current source at M_1_’s drain is provided by M_2_, which is a P-channel MOSFET with smaller transconductance.

The second stage is a common-source based current load, so that the drain current of M_8_ (I_bias_), is used to adjust the dc-gain of the amplifier. It utilizes a Miller Compensation combined with a custom feedback module for achieving good stability of the design. The stability of the feedback capacitor (C_F_) and the preamplifier open-loop gain determine the reliability of the preamplifier sensitivity. The open-loop gain is usually quite large, and hence the effect because of the small changes in the C_F_ can be ignored [39,40].

Therefore, the bias current is kept at a specific low value (2.5 µA) to keep a very low transconductance of M_3_ thus, exhibiting very high loop gain. Capacitor C_m_ provides gain and the dominant pole in that stage; so, a resistance R_m_ is used to suppress direct transmission through C_m_ at high frequencies [18]. Such a stage in the CSA incorporates a higher output resistance. All the transistors should be kept in their saturation state, i.e., V_GS_ > V_TH_ and V_DS_ > V_GS_-V_TH_ [6,39] to provide the maximum output swing for this stage. Here V_TH_ values for NMOS and PMOS are 0.6 V and −0.85 V, respectively.

The third stage consists of an N-channel MOSFET M_7_, which aims to give a negative gain of the entire circuit so that one can apply the negative feedback. It is biased by a low current through R_S_. The value of Rs is set to 3 kΩ so that M_7_ should operate in the saturation region. The output stage is source follower based, designed to exhibit unity voltage gain. Current flow from M_4_’s drain kept M_5_ biased in saturation. The feedback loop is built of an on-chip feedback capacitor C_F_ of 0.1 pF and an active resistor network M_F_-M_P_ of 3.54 MΩ and 1.42 kΩ, respectively, at the top-level design as shown in Figure 3. The circuit was designed with thick oxide transistors that allow a relatively high supply voltage of 3.3 V (VDD) in a standard 0.35 μm CMOS technology process. The achievable output rise time of the CSA circuit is given by $t_{r}=\frac{2.2}{2\pi \mathrm{GBW}}$, where GBW is the gain bandwidth of the CSA core amplifier. From this formula, a fast pulse response of 7.36 ns was guaranteed for reaching 1 GHz bandwidth.

### 2.2. Analysis of the CSA Circuit

The first stage is a cascade topology developed based on a common source with diode-connected PMOS (M_2_) so that the input is free from parasitic capacitance and the feedback amplifier controls the gate voltage.

**Figure 4:**
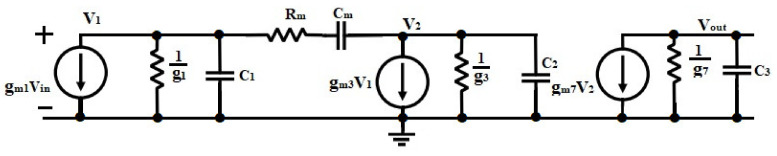
The small-signal model of the core Miller compensation with zero nulling resistors (MCNR) amplifier.

Therefore, the CSA input becomes a virtual ground and the sensor capacitance is less significant to the CSA bandwidth. The specifications of the design impose to guarantee a high dc-gain and high stability. The overall transfer function of the small-signal model of the proposed circuit (Figure 4) is presented as follows:

(1) A(s)=AOLDC1+CmRm+1gm3s1+gm3gm7r03ro8RsCmr03+ro8s1+Cmgm3+gm7gm3gm7s+CmCLgm3gm7s2

where $g_{mi}$, $r_{0i}$ and C_i_ are denoted as the equivalent transconductance, output resistance and the lumped capacitance at the ith gain stage. The output parasitic capacitance being lumped in the load capacitance C_L_. The parasitic capacitances and parameter values of the circuit in Figure 4 was extracted during the implementation process and presented in Table 2.

(2) $$\left\{\begin{array}{l} C_{1}=C_{gd1}+C_{gd2}\\ C_{2}=C_{gd3}+C_{gd8}+C_{gs7}\\ C_{3}=C_{gd7}+C_{gd6}+C_{L} \end{array}\right.$$

To study the stability of the design, the following assumptions are made to simplify the transfer function of the core amplifier. C_m_ and R_m_ being the Miller capacitor and the zero-nulling resistor, respectively, $C_{3}≅C_{L}\;and\;C_{m}\;,\;C_{L}$ >> $C_{1}$, $C_{2}$, $g_{mi}\gg \frac{1}{r_{0i}\;}$; thus, (1) can be written as

(3) $$A(s)=\frac{A_{OLDC}\left(1+\frac{s}{\omega_{z1}}\right)}{\left(1+\frac{s}{\omega_{po}}\right)\left(1+\frac{1}{Q}\frac{s}{\omega_{p1}}+\frac{s^{2}}{\omega^{2}{}_{p1}}\right)}$$

where the associated parameters are given by (4)

(4) $$\left\{\begin{array}{l} \omega_{z1}=\frac{g_{m3}}{C_{m}\left(1+R_{m}g_{m3}\right)}\\ \omega_{po}=\frac{r_{03}+r_{o8}}{g_{m3}g_{m7}r_{03}r_{o8}R_{s}C_{m}}\\ \omega_{p1}=\sqrt{\frac{g_{m3}g_{m7}}{C_{m}C_{L}}}\\ A_{OLDC}=-\frac{g_{m1}g_{m3}g_{m7}r_{03}R_{s}}{g_{m2}\left(1+\lambda r_{03}I_{bias}\right)}\\ Q=\frac{\sqrt{g_{m3}g_{m7}}}{\left(g_{m3}+g_{m7}\right)}\sqrt{\frac{C_{L}}{C_{m}}} \end{array}\right.$$

However, the dc-gain (A_OLDC_) of the circuit as depicted in (4) depends on I_bias_ and can be adjusted by an external resistor Rg; λ being the channel modulation parameter. (5) give the system’s phase margin (PM) with pole-zero cancellation

(5) PM=tan−1GBWωz1−tan−1GBWωpoQ1−GBWωpo2

**Figure 5:**
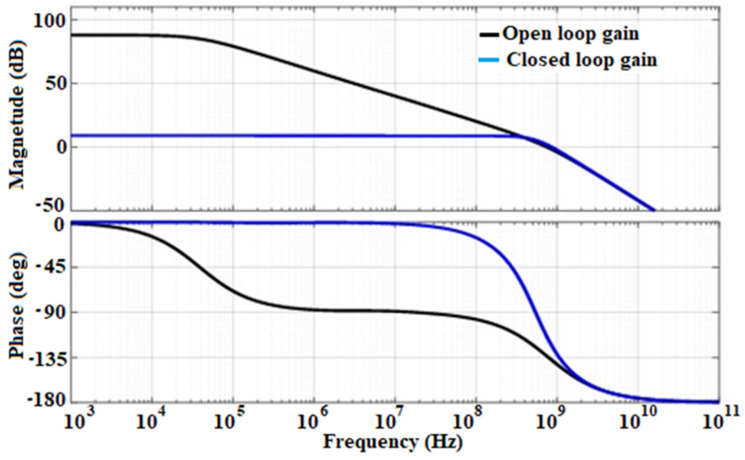
Graphical demonstration of the Open-loop gain (OLG) and Closed-loop gain (CLG) of the third-order system with a single-pole dominant pole.

The proposed circuit has been simplified and analyzed based on the MATLAB development toolkit [19]. Small-signal parameters and parasitic capacitances of MOSFETs are used in the toolkit as input data to enhance the design of multi-stage Opamps (Figure 5). Illustrates the frequency response of an MCNR three-stage Opamp designed for 42° phase-margin (black line). It is evident that the amplifier exhibited two poles; the dominant pole $\omega_{po}$, the large pole $\omega_{p1}$ and one zero, all associated with Equation (3). The poles are located at 74.6 kHz and 141.42 MHz, respectively, and the zero is situated at the frequency of 998 MHz. The feedback network is designed to introduce a phase lead near the crossover frequency, thus canceling the second pole of the Open-loop gain (OLG) which is located at the frequency of 141.42 MHz; then, increases the amplifier’s phase margin. The transfer function associated with the feedback network is written as (6):

(6) $$\left\{\begin{array}{l} K(s)=\frac{K\left(1+\tau_{F}s\right)}{\left(1+\tau_{p}s\right)}\\ \tau_{F}=R_{F}C_{F}\\ \tau_{p}=K\tau_{F}\\ K=\frac{R_{P}}{R_{P}+R_{F}} \end{array}\right.$$

As depicted in Figure 5, the Open-loop transfer function (OLTF) and Closed-loop transfer function (CLTF) are associated, respectively, with the Open-loop gain (OLG) and Closed-loop gain (CLG), the circuit should be designed to fit the requirements of this analysis. Therefore, rigorous transistor sizing and design should be implemented in order to achieve better performance, taking into account the parasitic effect and mismatch that generate noise in the device.

### 2.3. Feedback Lead Network (FLN) Implementation

This module comprises a charge collecting capacitor C_F_ and an active network resistor (MP and MF) based on a voltage-controlled NMOS resistor. The value of C_F_ was chosen to ensure sufficient high charge-gain conversion that will prevent the design against saturation. In fact, in most conventional CSA design, the charge gain is kept low enough to keep the preamplifier output from saturation. Since, the output saturation causes the ballistic deficit, which is a decrease in amplitude as the bandwidth has been degraded by the gain [35]. In this design, the bandwidth compensation is achieved thanks to the adjusted gain stage. Therefore, a feedback capacitor of 0.1 pF was set to handle a maximum input charge of 280 fC, without compromising the bandwidth. To minimize the feedback area, MP and MF are based on an NMOS transistor working in a linear region; their channel dimensions’ ratios are sized to exhibit no parallel noise. However, It is a challenge to bias the feedback network because to achieve a large effective resistance, the operating region of the MOSFETs is of interest. Considering a MOS device biased in strong inversion and working in the linear region, the drain-source current characteristics can be written as (7):

(7) $$I_{DS}=\mu_{n}C_{ox}R_{d}\left[\left(V_{GS}-V_{THN}\right)V_{DS}-\frac{V_{DS}{}^{2}}{2}\right]$$

Hence, M_P_ being biased to operate in the triode region, and neglecting the channel length modulation and the quadratic effect of the drain-source voltage, the equivalent resistor of the NMOS device is given as

(8) $$R_{DS}=\frac{V_{DS}}{I_{DS}}=\;\frac{1}{\mu_{n}C_{ox}\frac{W}{L}\left(V_{GS}-V_{THN}\right)}$$

M_P_ is designed to handle 1.42 kΩ equivalent resistor with ${\left(\frac{W}{L}\right)}_{P}$ = $\frac{2.772\;µ}{0.7\;µ}$. However, based on Equation (8), M_F_ was biased to operate in weak inversion moderate with ${\left(\frac{W}{L}\right)}_{F}$= $\frac{3\;µ}{36\;µ}$. This allows achieving a very large equivalent resistance of 3.542 MΩ.

### 2.4. Design of the CR-RC^1^ Pulse Shaper

**Figure 6:**
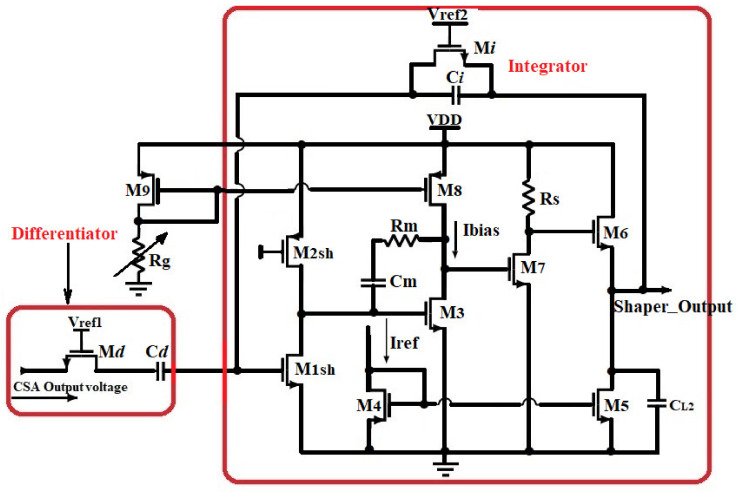
Schematic of the proposed structure of the Shaper.

In order to tune the signal-to-noise ratio (SNR) of the sensor readout electronics and reduce the signal interference between signals from a different time, the output signal of the CSA is needed to be shaped using a first-order active CR-RC pulse shaper (PS) circuit as illustrated in Figure 6. Low-frequency noise (1/f) and thermal (high-frequency) noise was suppressed using a custom shaper circuit consisting of a differentiator and an integrator with constant time both equal to the optimal shaping time ($\tau_{d}=\tau_{i}=\tau_{s,opt}$). The pulse shaper circuit provides an output voltage proportional to the energy of the detected particles. The topology of the core amplifier used in the CSA is used for this purpose. Therefore, the loop gain $A_{\mathrm{OL}\_\mathrm{SH}}$ of the PS is given by (9) as follows:

(9) $$A_{OL\_SH}=\frac{∆V_{CSA,max}}{∆Q_{max}}C_{F}{\left(\frac{e}{n}\right)}^{n}n!$$

where n is the order of the shaper. Using the design parameters allows achieving 2.67 loop gain. It is easy to derive the shaping design parameters as follows: $C_{d}R_{d}$ = $C_{i}R_{i}$ and $\frac{R_{d}}{R_{i}}$ = $\frac{C_{d}}{C_{i}}=\frac{1}{A_{\mathrm{OL}\_\mathrm{SH}}}$. For 200 fF integrating capacitor, $C_{d}=534\;\mathrm{fF},{\;R}_{d}=$ 400.75 kΩ and $R_{i}=$1.07 MΩ, respectively.

Henceforth, $R_{d}$ and $R_{i}$ are very large, thus should occupy more space. Using (8) with suitable transistor biasing within the design process, the equivalent resistance can be derived from NMOS device operating in weak inversion moderate so that $\frac{W_{i}}{L_{i}}=\frac{10\;µ}{41\;µ}$, $V_{\mathrm{GSi}}=0.7\;V$ and $\frac{W_{d}}{L_{d}}=\frac{2\;µ}{23.6\;µ}$, $V_{\mathrm{GSd}}=$ 0.9 V. However, the PS core amplifier would exhibit a gain-bandwidth given by ${\mathrm{GBW}}_{\mathrm{SH}}=\frac{1}{2{\pi \tau }_{s,\mathrm{opt}}}=744.1\;\mathrm{kHz}$. Hence, ${\mathrm{GBW}}_{\mathrm{SH}}=\frac{g_{m1\;\mathrm{sh}}}{2{\pi C}_{L2}}$, $g_{m1\mathrm{sh}}$ being the transconductance of the input transistor and $C_{L2}$ the total load capacitance of the shaper. For 1 pF, load capacitance, the small-signal transconductance is calculated from the previous expressions and controlled to be 4.67 μS, which allow simulating 912 nA drain-source current, exhibiting, therefore, the ultra-low-power dissipation of only 0.301 µW, while the geometric aspect ratio of the device was controlled at $\frac{W_{\mathrm{sh}1}}{L_{\mathrm{sh}1}}=\frac{3\;µ}{20\;µ}$. Moreover, the shaper input stage was chosen to be a common source with a P-channel MOSFET active load. The former device was biased to work in a strong inversion saturation regime by $V_{b}=1.2\;V$, and adjusted to handle $A_{\mathrm{OLSH}}=-10$ input gain stage; so $g_{m2\mathrm{sh}}=\frac{g_{m1\mathrm{sh}}}{10}$. Despite the input transistors M_1sh_ and M_2sh_ which have been customized, the remained devices of the CSA core amplifier have been utilized to design the shaper module. The general parameters of the PS circuit are presented in Table 2. 

### 2.5. Noise Optimization of the FEE Circuit

The sensors, preamplifiers and shapers are the main contributors to noises. The CSA, along with providing low-noise amplification, offers low input impedance (virtual ground) which stabilizes the potential of the sensor electrode and reduces the inter-electrode cross-talk [41]. The input transistor of the CSA is designed to operate in strong inversion saturation and optimized to handle the lowest possible ENC. The total $ENC_{CSA}$ for a given feedback and sensor capacitor, according to the adopted CMOS process consist of three different components [6] and given as follows:

The most prominent thermal noise contribution can be calculated as (10):

(10) $$ENC_{th}{}^{2}=\;\frac{4K_{B}Tn\gamma \alpha_{n}}{q^{2}}\frac{{\left(C_{det}+C_{f}+\;C_{g}\right)}^{2}}{g_{m}C_{g}}\frac{N_{th}}{\tau_{s}}$$

where *K_B_* is the Boltzmann constant, *T* is the room temperature, *η* is the body factor, γ is the inversion factor, α_n_ the excess noise factor, *N_th_* is the shaper noise index for the thermal noise, *τ_s_* is the peaking time, *C_det_* the sensor capacitance, *C_f_* the feedback capacitor, *C_g_* the gate capacitance and $g_{m}$ is the input MOSFET transconductance.

The flicker noise also known as 1/f noise is expressed as (11):

(11) $$ENC_{1/f}{}^{2}=\;\frac{K_{f}}{q^{2}}\frac{{\left(C_{det}+C_{f}+\;C_{g}\right)}^{2}}{C_{g}}N_{f}$$

where *K_f_* is the flicker noise coefficient and *N_f_* the shaper noise index for flicker noise

The white parallel noise contribution due to the sensor leakage current (*I_leak_*), the MOSFET gate current and feedback resistor $R_{f}$, is defined as follows (12):

(12) $$ENC_{i}{}^{2}=2q\left(I_{leak}+\;I_{G}\right)N_{i}\tau_{s}+\;\frac{4K_{B}TN_{i}}{q^{2}R_{f}}$$

where *q* is the elementary charge, $I_{G}$ the gate current of the input transistor, $R_{f}$ the feedback resistance and $N_{i}$ the shaper noise index for the white noise. In Equation (12), the first term refers to shot noise for a weak inversion MOSFET operation due to a higher potential barrier between source/drain and channel. However, the second term refers to the thermal noise generated by the very small potential barrier created by the positive gate potential in a strong inversion MOSFET [7,25]. 

Different components of the ENC were first optimized with respect to *W* and *I_D_*, and then with respect to $C_{g}$ [6] using the first-order shaper. The optimization technique well explained in refs. [6,33] is therefore adopted and the optimized parameters are derived as follows $W_{opt}=\frac{3\left(C_{det}+C_{f}\right)}{2C_{ox}L_{min}}$ and $I_{D,opt}=\frac{g_{m}{}^{2}L_{min}}{2\mu_{n}C_{ox}W_{opt}}$. The instability of the drain current (*I_D_*) is established by the variation of charge in the depletion region, which constitutes the channel width. $L_{min}$ and $W_{opt}$ are the minimal length and the optimal width of the input device. $W_{opt}$, being calculated at 62.5 μm and $L_{min}=10.5\;µm$ the design requirements allow achieving very much less drain current of $I_{D,opt}=2.5\;µA$, for the CSA input transistor. Since the bias current of M1 is fixed to its optimal value, increasing W/L reduces the overdrive voltage *V_GS_*-*V_TH_*, eventually driving the transistor in moderate or weak inversion. The threshold voltage variations were reduced based on conventional low-threshold voltage (LVT) operation, which consists of lowering channel doping, which narrows the channel depletion region, improves the subthreshold slope, and reduces the gate leakage contribution. Moreover, the V_TH_ optimization was implemented during the Spice simulations setting the bulk-source voltage of the inputs transistors to 0 (V_BS_ = 0 V). Moreover, while layout the design, mismatch reduction helps in reducing the fluctuation of V_TH_ taking into account the trade-off between drain-induced barrier lowering (DIBL) mitigation and gate leakage reduction [42]. Therefore, if the transistor works in this region, increasing its gate width too much worsens the noise, because it leads to more gate capacitance without improving the transconductance [6,41,43]. The total gate capacitance, which optimizes the different components of ENC, is obtained by solving the equations $\frac{\partial ENC_{th}{}^{2}}{\partial C_{g}}=0$ and $\frac{\partial ENC_{1/f}{}^{2}}{\partial C_{g}}=0,$ respectively [6]. The solutions of those equations are found to be:

(13) $$C_{g,th}=\frac{3}{2}\left(C_{det}+C_{f}\right)\;\mathrm{and}\;C_{g,1/f}=\left(C_{det}+C_{f}\right)$$

The values of the gate capacitances given by (13) limit the operating regime of the input device. The gate width is finally adjusted to achieve the matching condition defined in (13). At this point, if the contribution of the ENC due to flicker noise is greater than the one given by thermal noise, *Cg* can be further increased. Depending on the value of *K_f_* and the peaking time, the optimization will result in a *W* yielding a gate capacitance between $\frac{3}{2}\left(C_{det}+C_{f}\right)$ and $\left(C_{det}+C_{f}\right)$. The input capacitance must also be much greater than the other capacitance sources connected to the input preamplifier in order to ensure that the sensitivity of the preamplifier is not compromised by external capacitance changes [25]. Considering the input transistor in the strong inversion saturation mode, $W_{opt}$ leads to $C_{g,opt}=\left(C_{det}+C_{f}\right)$. Thus, in this regime, the same value of gate capacitance minimizes both flicker and thermal noise. Therefore, the total ENC of the CSA can be expressed as (14):

(14) $$ENC_{CSA}=\frac{1}{q}\left(\left(\sqrt{\frac{A_{1}}{g_{m1}\tau_{p}}}+\sqrt{A_{2}}\right)\left(C_{det}+C_{f}\right)+\left(q\left(I_{leak}+\;I_{G}\right)\tau_{s}+\;\frac{4K_{B}T}{R_{F}}\right)N_{i}\right)$$

where $A_{1}=\;\frac{K_{B}Tn\gamma \alpha_{n}}{3}N_{th}$ and $A_{2}=\;\frac{16K_{f}N_{f}}{3}$. 

However, the passive feedback resistance ($R_{f}$) is replaced by the voltage-controlled NMOS resistor network, which exhibited no parallel resistive noise. Moreover, the optimal shaping time is obtained by solving the equation $\frac{\partial ENC_{total}{}^{2}}{\partial \tau_{p}}=0$. Thus (15) give optimal shaping time and (16) give the optimized ENC as

(15) $$\tau_{s,opt}=\sqrt{\frac{A_{1}}{qI_{leak}g_{m1N_{i}}}}\left(C_{det}+C_{f}\right)$$

(16) $$ENC_{CSA}=\frac{1}{q}\left(\left(\sqrt{\frac{A_{1}}{g_{m1}\tau_{s,opt}}}+\sqrt{A_{2}}\right)\left(C_{det}+C_{f}\right)+\left(q\left(I_{leak}+\;I_{G}\right)\tau_{s,opt}+\;\frac{4K_{B}T}{R_{F}}\right)N_{i}\right)$$

From analytical computation, it is clear that the minimum ENC of the CSA is achieved when $\tau_{s}=$214 ns, which is the shaper constant time.

Assuming that the sharper module exhibits infinite gain and higher SNR, the impact of noise from its amplifiers can be reduced by increasing the size and power of the active devices [6,40]. The ENC contribution of the shaper comes from the dissipative feedback component [6]. The parallel noise spectral can be stated as an equivalent parallel noise generator at the input of the charge amplifier by scaling it with the square of the charge gain of the shaper $A_{OL\_SH}$ [6,41,44,45]. Thus, the shaper ENC component is given as (17):

(17) $$ENC_{SH}{}^{2}=\frac{4K_{B}T}{A_{OL\_SH}{}^{2}R_{i}}N_{p}\tau_{s}$$

where $N_{p}$ is the ENC coefficient for white parallel noise [34].

The total ENC of the FEE, defined as the quadratic sum of the CSA and the shaper components can be expressed by (18) as follows:

(18) $$ENC_{total}=\sqrt{\begin{array}{c} \frac{1}{q^{2}}{\left(\begin{array}{c} \left(\sqrt{\frac{A_{1}}{g_{m1}\tau_{s,opt}}}+\sqrt{A_{2}}\right)\left(C_{det}+C_{f}\right) \end{array}\right)}^{2}+\left(q\left(I_{leak}+I_{G}\right)\tau_{s,opt}+\frac{4K_{B}T}{R_{F}}\right)N_{i}+\frac{4K_{B}T}{A_{OL\_SH}{}^{2}R_{i}}N_{p}\tau_{s,opt} \end{array}}z$$

## 3. Simulation Outcomes and Discussions

### 3.1. Simulation and Implementation Framework

The performances of the proposed readout circuit were verified using LTSpice simulator and the layout was implemented in 0.35 µm CMOS technology process from TSMC, using Electric VLSI. For all the Spice simulations, the sensor was modeled by an ideal current source in parallel with capacitor C_det_ which values vary up to 2 pF. The Transistors were placed symmetrically, biased and designed by keeping the ratio $\frac{g_{m}}{I_{D}}$ sufficiently high in order to optimize mismatch along with the stability of other analog performance such as the gain-bandwidth product GBW [41,43]. The CSA input’s transistor size and biasing current were optimized for matching the input capacitance to the target sensor’s capacitance [26]. It was, therefore, biased with a low current of 2.5 µA supplied from 3.3 V (VDD). The shaper’s core is based on a common source input stage with a P-channel MOSFET active load, biased to work in a strong inversion saturation regime with $V_{b}=1.2\;V.$ This allowed simulating 912 nA drain-source current, exhibiting, therefore, an ultra-low-power dissipation of only 0.301 µW and achieving the GBW of 744.1 kHz. Its peaking time was configured optimizing the overall ENC of the FEE and controlled at 214 ns.

### 3.2. Results and Discussions

**Figure 7:**
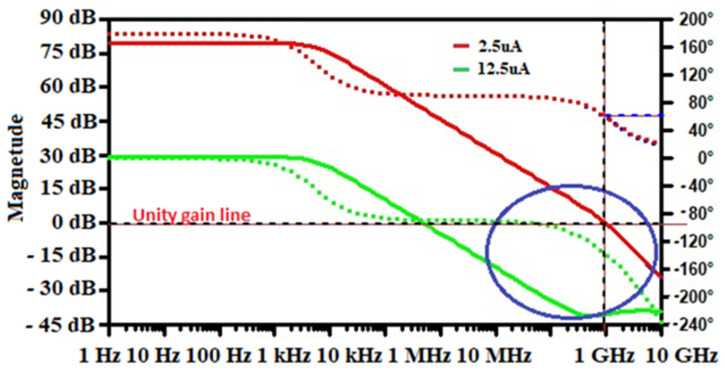
Influence of bias current (I_bias_) on the Open-loop gain.

**Figure 8:**
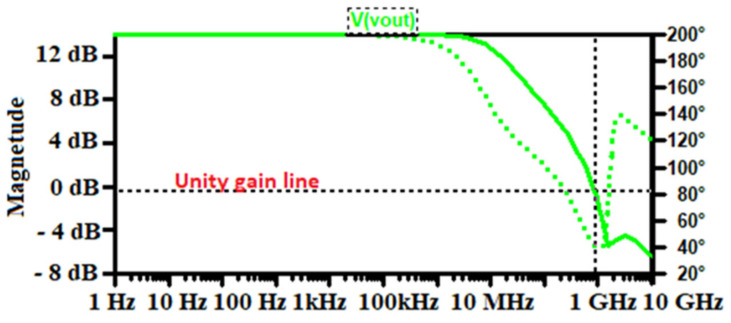
Bandwidth compensation using feedback lead network-based a MOSFET resistor; which allows achieving high stability.

The specifications and design parameters of the proposed front-end electronics were improved as compared to recently published works. Figure 7 shows the influence of the bias current on the open-loop gain of the core amplifier. As illustrated in that figure, is possible to increase the dc-gain of the device just by adjusting I_bias_ value, for a feedback loop of R_F_ = 3.542 MΩ and C_F_ = 0.1 pF. To achieve suitable amplification of the CSA, I_bias_ was controlled to 2.5 µA by an external resistor (Rg) as mentioned in the previous section. Frequency analysis swept from 1 kHz to 10 GHz and is displayed in decade form. The bias current is adjusted by changing the value of the external resistor Rg that allows changing the transconductance of M8, and therefore increasing the dc-gain of the Opamp as depicted in Equation (4). Figure 7. shows the Spice simulation results of the open-loop gain (OLG) of the Opamp versus the I_bias_ current. It is evident that for the low value of I_bias_, wide GBW is achieved but involves poor stability of the circuit. The simulated results show that the core amplifier achieved a 2.5 µA bias current, a unity gain-bandwidth of 997.84 MHz with a 42° phase margin. The very little difference with the analytical value is due to the parasitic and the residual noise generated by the circuit. However, the phase margin remains poor and the circuit behaves unstable. Therefore, the bias current is a crucial parameter that may guarantee high dc-gain, the stability of the circuit need to be compensated. Since the GBW is stabilized through the dc-gain, it should be necessary to keep the highest possible phase margin for maintaining signal integrity [23,24,46]. Therefore, its feedback network determines the closed-loop gain (CLG) stability of the design. Since the sensor, the capacitance was set to 2 pF and the extracted parasitic capacitor of the input transistor was around 20 fF; the total input capacitor was fixed to 2.02 pF. Nevertheless, a resistor has a parasitic capacitance and a capacitance has a parasitic resistance. Thus, an RC feedback network (R_F_-C_F_) models the feedback circuit. Loop-gain stability has been tested during the charge vs voltage conversion when R_F_-C_F_ is bypassed [21]. The Opamp equivalent load capacitors are also taken into consideration by varying C_F_. For achieving the highest stability of the circuit, the closed-loop gain is adjusted by the R_F_-C_F_ sizing. The feedback equivalent resistor (R_F_) was implemented by associating the drain-source resistance of two N-channel MOSFETs (M_F_ and M_p_ on Figure 3) device biased to be in the triode strong inversion region. Under this condition, the parallel noise was minimized to a large extent; thus, the circuit is stable and continuously sensitive and can be maintained in this condition without adjustment for spectroscopy purposes [16,40,41,43]. Thus, with that technique, we achieved up to 3.542 MΩ feedback equivalent resistances, which guarantee a phase margin of 82°. The closed-loop gain of the design is shown in Figure 8. As depicted on that plot, the maximum unity bandwidth (GBW) achieved by the design (for stability conditions) is controlled at 1 GHz, which is a bit different from the one obtained in the open-loop condition. Thus, the feedback compensation circuit and the parasitic capacitance of the design produce an error estimated at 0.216% on the GBW. The difference between the analytical model is just 0.016%. This little difference is because the analytical solution was computed with ideal components, neglecting, therefore, some internal capacitance and mismatch produced by the devices. Adjusting I_bias_ as shown in Figure 7, enhances the phase margin and the bandwidth could be extended to more than 2 GHz. The compensation capacitor brings together a pole and zero into the loop equation. The zero always occurs before the pole because of R_F_ > R(M_F_)||R(M_p_). The zero is placed to cancel out the first pole along with its associated phase shift. The analytical closed-loop transfer function shown in Figure 5 (blue line), was confirmed by the Spice simulation results in Figure 8. When the $\tau_{F}$ zero is placed at ω_p1_, it cancels out the pole (p1) causing the Bode plot to continue on a slope of −20 dB/decade. When the frequency gets to ω_F_ = 1/R_F_C_F_, this pole changes the slope to −40 dB/decade. The phase shift is canceled before the second op-amp pole occurs, and the circuit reacts as if the pole was never introduced. The benefit of pole-zero cancellation is improved pulse shape and resolution in the energy at a high counting rate [4,23,25,32]. 

**Figure 9:**
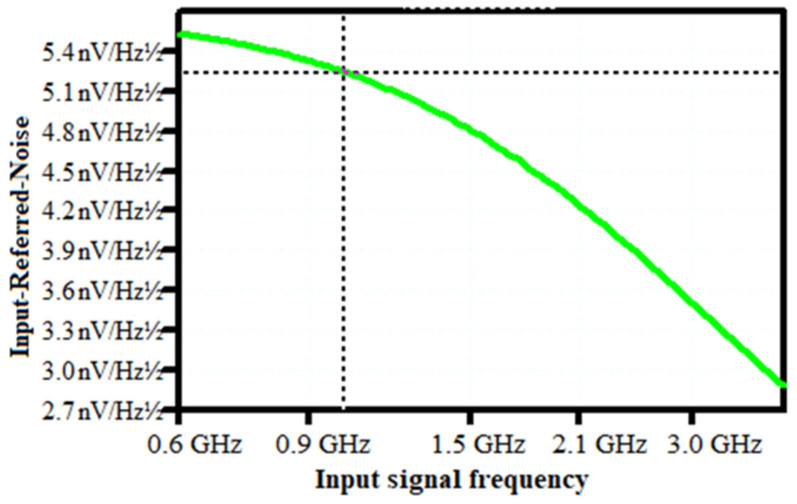
CSA Input-Referred Noise.

**Figure 10:**
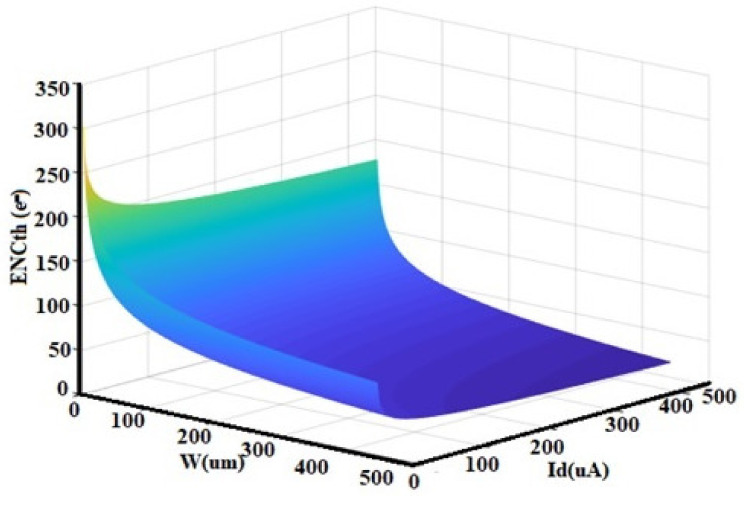
ENCth as a function of W and drain current (Id) [6].

The noise corner frequency fc, which is the frequency at which the asymptotes of the flicker and thermal noise components cross was identified as the frequency range over which the CSA op-amp noise is dominated by either the 1/f or the thermal noise components [36,37,38,39,40,41,42,43,44,45,46,47,48,49]. In agreement with this definition [36], the noise corner frequency of our design has been controlled to be 652.9 MHz. Therefore, the Input-referred-noise (IRN) of the circuit was plotted in Figure 9, in the frequency range of 600 MHz to 4 GHz. The IRN spectral density extracted is 5.23 nV/√Hz at 997.82 MHz. Moreover, when developing analog front-end recording (AFE), a lower IRN guarantees the signal quality [16] of the recorded neuron activity and low power consumption can prolong the existence of the implanted recording system in the human body [6,36]. However, in the CSA, the parameter that embodies the noise performance is the ENC, namely the input charge necessary to get at the output a signal equal to noise. Its calculation was based on this intrinsic definition, neglecting the standard calculation depending on the post-CSA circuit, not present in this design [33,34,36]. Equations (18) and (19) have been computed to provide optimal design parameters; an optimum shaping time of 214 ns has been extracted and the overhead ENC has been controlled at 37.35 e-with a sensor capacitance of 0 pF and a slope of 16.32 e-/pF worsened the noise; while the Spice simulations provided a noise slope factor of 19.58 e-/pF. The ENC as a function of I_D_ and W has been computed and presented in Figure 10. It is clear that the thermal noise is decreased when an increase in the input transistor current occurs but it comes up with the increase in the bandwidth over which the thermal noise is integrated as well. Therefore, those effects canceling each other out. Hence, significant reductions in power consumption are achievable with little or no noise penalty if the device is made to operate at a low count rate [46,47,48]. Moreover, the reduction in the bias current of the input transistor offers good separation between the preamplifier rise and fall time [17,48,50,51]. According to Equation (19), we can note that, at short peaking times, the noise increases rapidly with capacitance and increases as the peaking time is reduced. For Si-PIN diodes, the capacitance scales with area, so large area sensors exhibit more noise [12,37,38].

**Figure 11:**
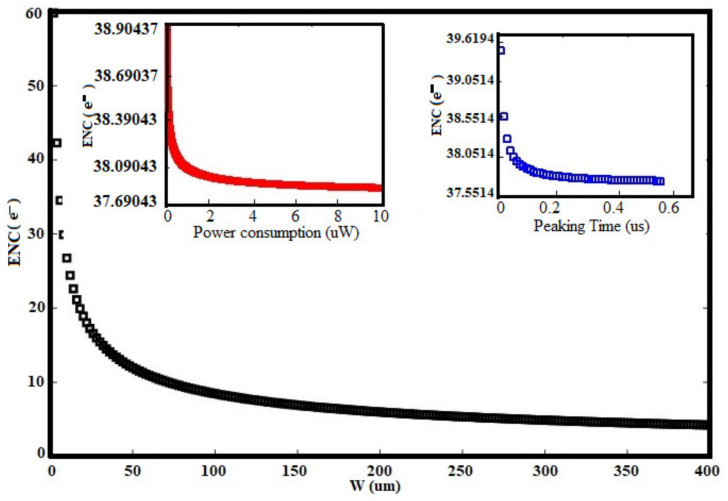
Equivalent Noise Charge (ENC) as a function of W.

For SDDs, the capacitance is much lower and nearly independent of area. This noise is only weakly dependent on temperature [12,37,43]. At long peaking times, the noise increases with peaking times (Figure 11) and with leakage current. Since leakage current increases exponentially with temperature, reducing temperature helps dramatically.

**Figure 12:**
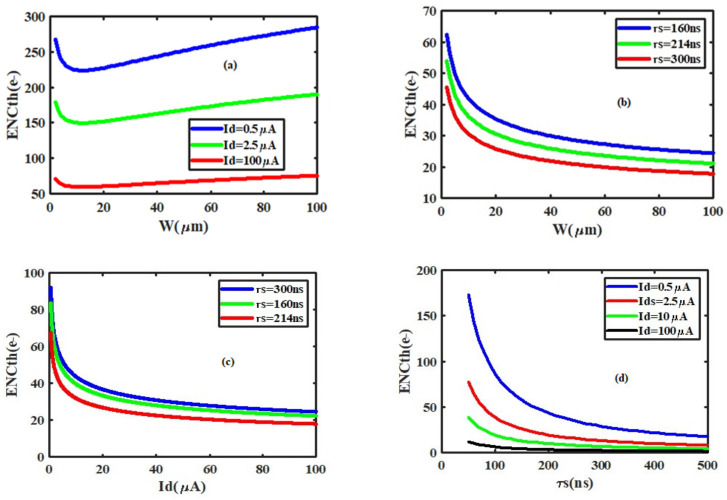
ENCth versus different design parameters: (**a**) as a function of W for different sets of the input device drain current; (**b**) as a function of W for different sets of the shaping time; (**c**) as a function of the input device drain current for various peaking time; (**d**) versus the peaking time for different sets of the input transistor drain current.

There will be always some peaking time at which the noise is minimum, where the delta and step terms are equal. There is no advantage for operating at a longer shaping time, because of the integration of more parallel noise during this period. The optimum time constant is shorter for lower capacitance and longer for low leakage currents. Otherwise, the third term of Equation (14) represents the shot noise (due to the leakage current of the sensor) which could be considered to be 10 nA (for the worst silicon sensor) while performing the total noise of the intrinsic CSA circuit (Figure 11). The intrinsic noise represents the noise of the preamplifier without any sensor connected. The ENC varies from 39.0437 e^−^r.m.s to 37.5643 e^−^r.m.s as the peaking time is changed from 10 ns to 0.5 µs. From the spice simulations, it is shown in Figure 11 that, the ENC is reduced when the power dissipation increases. The ENC achieves a value of 37.69043 e^−^rms when the power dissipation is larger than 8.56 µW. This means that the specification of the power dissipation satisfies the design requirements. As shown in Figure 12a–d, the design consideration taken to optimize the total ENC for the used techniques is also used to choose the optimal Id and W, as a trade-off between ENCth specification of the peaking time and power consumption [4,16,25]. Those optimal parameters were Id = 2.5 µA and Wopt = 62.5 µm which corresponds to gm = 61.4 µS. An optimal transistor channel length Lmin = 10.5 µm was chosen to minimize the input capacitance of the CSA circuit, therefore. Especially on Figure 12a,b), it is evident that ENC_th_ has a minimum value at Wopt, and that value has a low dependency on Id and $\tau_{s}$, respectively. From those two graphs, it is clear that above 62.5 µm the noise improvement with the drain current and the peaking time increasing, respectively, is very low. The same observations are made in Figure 12c where the dependency of the ENCth is very low above 2.5 µA.

**Figure 13:**
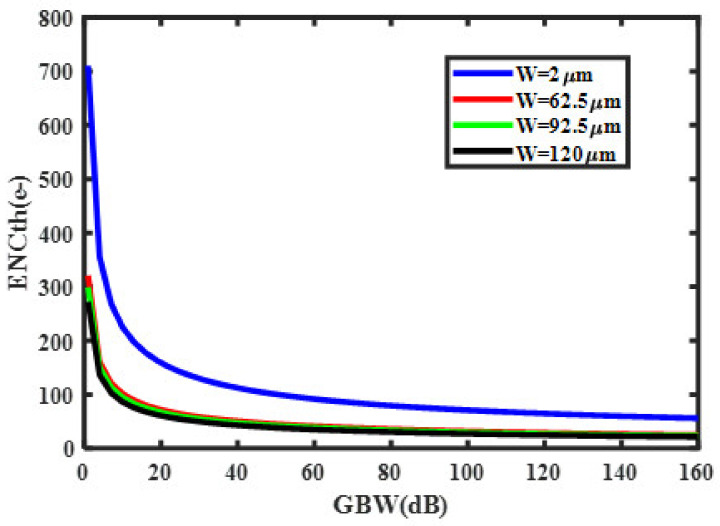
Effect of the CSA gain bandwidth (GBW) on ENCth for the different input gate widths.

**Figure 14:**
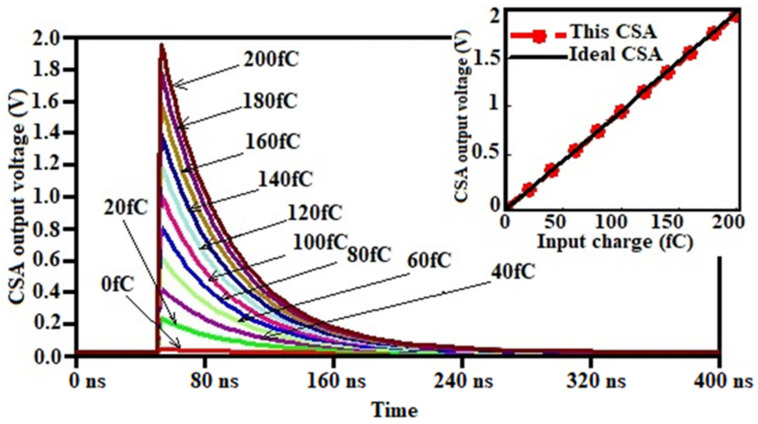
CSA output voltage for different input charge.

**Figure 15:**
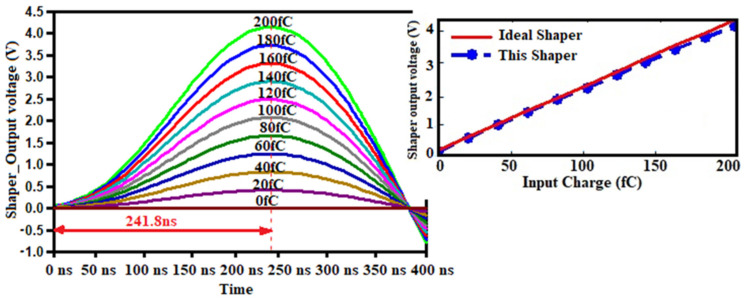
Pulse shaper (PS) output voltage for different input charge.

The transient responses of the readout circuit are shown in Figure 13 and Figure 14. Different charges of width 1 ns were injected into the sensor. The output swing of the CSA achieves up to 1.962 V peak and decreases slowly thereafter because of the feedback action. The fall time of the signal is about 300 ns, setting by C_F_ and R_F_. It is evident in Figure 14 and Figure 15 that the CSA output is amplified and shaped; for 200 fC-injected charges, the shaper output swing achieved the peak value of 4.16 V after 241.8 ns peaking time.

The input charge dynamic range of the FEE is from 0 fC to 280 fC. The output voltage linearly increases with the increase of input charges, the charge-to-voltage gain from the output node of the CSA, the CR-RC shaper, is provided by simulation outcomes as, 546.56 mV/MeV (9.92 mV/fC) and 920.66 mV/MeV (16.7 mV/fC), respectively, using the equivalence from mV/fC to mV/MeV as mentioned in ref. [52]. The output voltage range of the Shaper is 22 mV to 4.16 V. The overall gain of the readout module can be adjusted by the feedback capacitance of the CSA.

Figure 13 shows the effects of the CSA gain bandwidth on the ENC_th_, with different input transistor widths. It is readily recognized that the lower transistor width leads to higher thermal noise for GBW from 1 to 20 dB. This is because, for lower GBW, the collection process is slowed down; due to the highest rise time, the thermal noise accumulated in the device increases accordingly. This results in the attenuation of the output swing and therefore a poor energy resolution [4,25,34]. As depicted in Figure 13, the optimal input transistor width (62.5 µm), is the critical value for which the variation of the thermal noise is not sensitive to the CSA gain bandwidth. Therefore, from a point of view of minimizing the ENCth, a typical gate width is needed at a higher GBW [4,25]. From a practical point of view, higher GBW leads to a short rise time than a very fast collection process. So, instead of the wide bandwidth of the CSA, the noise accumulation process is very brief due to the shortest collection time (7.36 ns) [5]. Accordingly, the optimal input noise matching results in an optimum input device aspect ratio. The smallest transistor size should be therefore taken at the expense of some system resolution [4,25,34]. However, the output stage of the shaper being an N-channel source follower will help in reducing the non-linearity of the device for the large output signal. The nonlinearity of the readout module was controlled at only 0.8% and 1.24%, respectively, for the CSA and shaper, provided by the spice simulation results.

**Figure 16:**
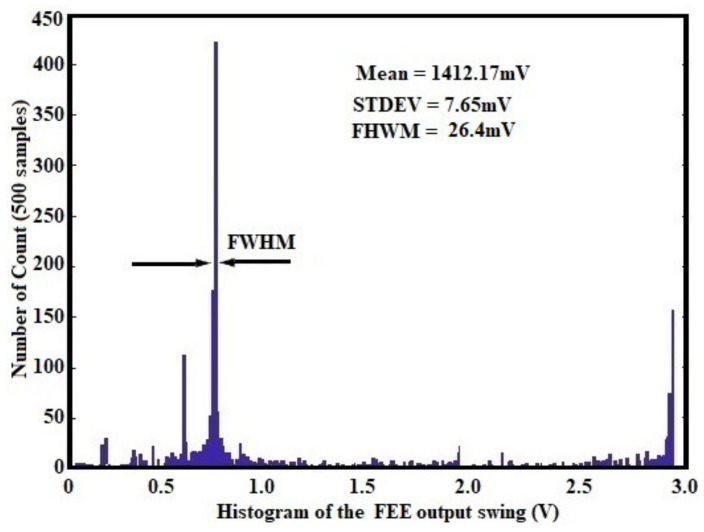
Histogram of output voltage for high charge production rate.

The capacity of the circuit to operate under high particle flux and high charge production rate was simulated and presented in Figure 16. The sensor with 2 pF capacitance was set to handle 1000 radiation events. Up to 280 fC charges were therefore injected at preamplifier input with 1 fC maximum step. The output swing of the circuit was computed and the histogram of the amplitude was therefore generated.

### 3.3. Post Layout Monte Carlo Simulation Results

**Figure 17:**
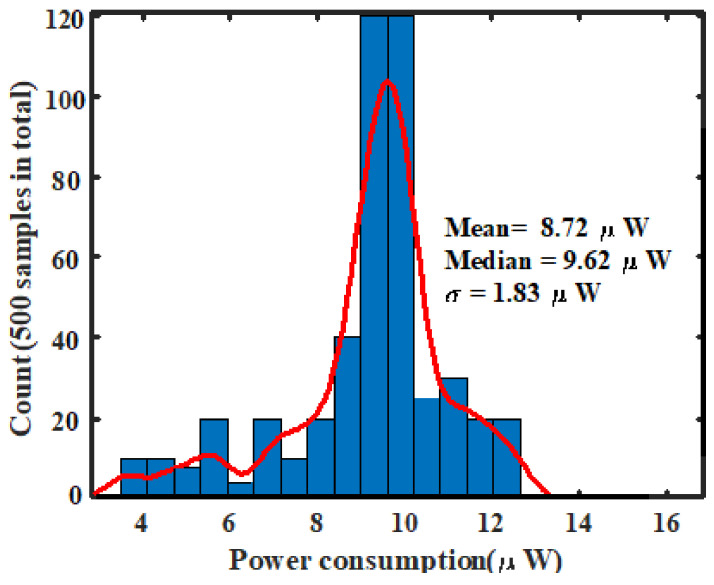
Histogram of power consumption against process variation.

Power-efficiency and robustness of the proposed circuit against process variation were performed through a post-layout Monte Carlo simulation. As illustrated in Figure 16, this histogram describes the response function of the proposed FEE against several radiation events. This corresponds to the histogram of the energy of the detected particles (or injected charges) in real-time operations [6,8,37,47]. Two important observations can be made. On the one hand, the output swing (offset voltage) for 0 fC is very low and is about 22 mV. This means that the proposed FEE does not exhibit high input offset; this confirms the zero dc-voltage components shown in Figure 14 and Figure 15 for different input charges. The radiation-hardened behavior of our proposed front-end has been achieved thanks to input transistor sizing which helps in keeping lower gate capacitance and optimal transistor width for a considerable reduction in electric noise [9,12]. On the other hand, the proposed design is capable of handling up to 280 fC without losing the integrity of the signal (preserving the information of interest). So, exhibited a wide input charge range. The mean output swing of the design was controlled at 1412.17 mV with a 7.65 mV standard deviation. The full-width half-maximum (FWHM) was only 12.23 mV and contributed only at ~1.87% of the output swing. Since the circuit energy response is illustrated by Figure 16, the lowest percentage of the FWHM is satisfactory and confirms that the proposed FEE can handle high-energy resolution [12,16] for spectroscopic applications. In Figure 17 the post layout Mont Carlo simulation results, highlighting the ultra-low power behavior of our circuit is presented. The average power consumption of the design was controlled at 8.72 µW while exhibiting only 1.83 µW of standard deviation. From this analysis, it can be concluded that, the power dissipation of the proposed front-end does not vary significantly due to process variations.

**Figure 18:**
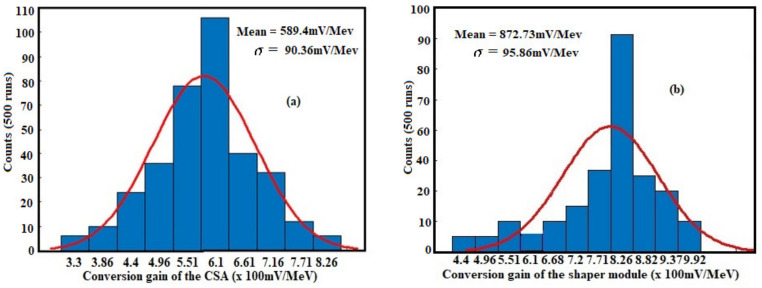
Histograms of the conversion gain for both (**a**) the CSA and (**b**) the PS.

Figure 18 shows the histograms of conversion gain based Monte Carlo simulation results of the proposed front-end circuit for 500 runs, which exhibited the histogram of the conversion gain for both the CSA circuit and the PS module, for 10 fC charge injected at the input of the sensor. The highest sensitivity of the design is then achieved; for a week amount of injected charge the histograms of the conversion gain observed on Figure 18a,b show a mean value of 589.4 mV/MeV, and a standard deviation of 90.36 mV/MeV for the CSA stage while the shaper circuit exhibited 872.73 mV/MeV mean value and 95.86 mV/MeV standard deviation. This shows that the outcomes got with Monte Carlo models do not vary fundamentally for 500 runs and the front-end performance is very steady and robust. The less difference of those parameters with the spice simulation results is attributed to the parasitic capacitance obtained while designing the feedback circuits of the different stages. This can be compensated by adjusting the feedback capacitance of the CSA or increasing the loop gain of the shaper via an external device.

**Figure 19:**
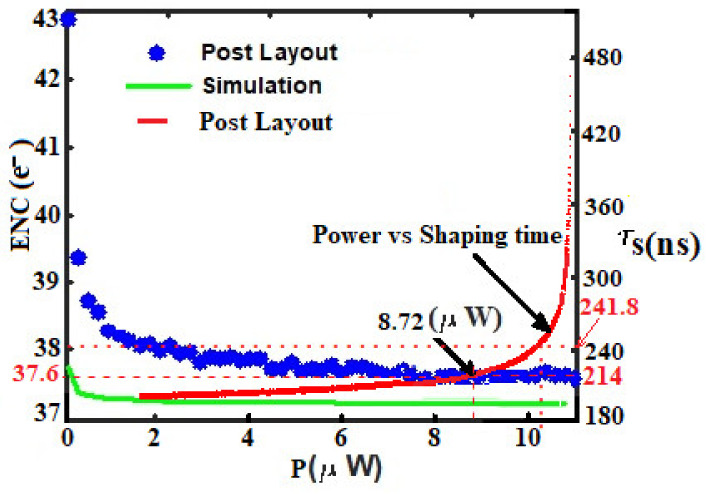
Validation of the design performance in terms of ENC, power consumption, and shaping time.

Moreover, as highlighted in Figure 19, the ENC and shaping time are extracted from the post-layout simulation results and plotted for different values of power consumption. The system achieved an ENC of 37.6 e^−^ at 214 ns peaking time while dissipating only 8.72 µW of power from 3.3 V supply voltage. At 241.8 ns peaking time, the proposed front-end exhibited an ENC of 38 e^−^, while consuming very less power of 10.14 µW. Those relatively low variations of equivalent noise charge and power consumption provided by the post-layout simulation at 241.8 ns peaking time, do not differ so much from those provided by the spice simulations; confirming, therefore, the ultra-low-power and low-noise behavior of our design.

**Figure 20:**
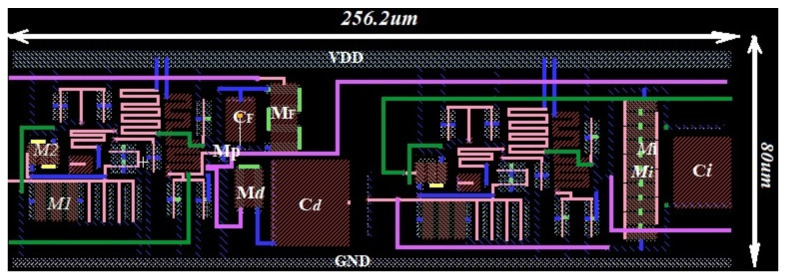
Core layout of the proposed readout FEE.

The total core layout area occupied by the proposed readout electronics is sized at (256.2 × 80) μm^2^ as shown in Figure 20. Parasitic extraction was used to extract the netlist with parasitic. The voltage supply is 3.3 V; the maximum power consumption achieved through post-layout simulations is about 8.72 µW for the whole circuit, which is 1.83% higher than that provided by the spice simulations. This little increase in power dissipation is mostly due to the parasitic and mismatch while laying out the design [11,17,49]. In this research, the gain-bandwidth product of the circuit was stabilized by means of a high-frequency feedback loop, which operates according to the voltage-controlled NMOS resistor (R_F_ and Rp) technique [6,22]. The innovation of the proposed FEE results in the implementation of the external bandwidth compensation based gain stage, which allows achieving high gain with less amount of current, preventing, therefore, the pulse height degradation along with bandwidth limitation and power dissipation. Further, the combination of the Miller compensation with the Feedback lead network is used to raise the best PM and guarantee decent stability of the gain-bandwidth product with good linearity for high-energy resolution applications.

As a rundown, in Table 3 the general highlights of the FEE circuit are presented. To achieve a high signal-to-noise ratio (SNR) and reduce power consumption, ENC, and active die area of the chip, the configurations presented in the literature have been consulted [6,14,16,20,22,24,39,52,53,54,55,56]. Considering the critical contrast on the input transistor’s capacitance, the outcomes are empowering. Therefore, readout electronics performances are in agreement with the state-of-art specifications. On the one hand, the design of the input and feedback transistors allowed achieving high linearity, with high phase margin and sufficient low noise to ensure good stability. On the other hand, the optimization of I_bias_ helps in adjusting the dc-gain of the CSA circuit and avoids saturation, which affects the linearity and the energy resolution of the device. Therefore, the adjusting gain stage allows achieving a high-energy resolution with wide gain bandwidth (1 GHz) and the operational amplifier stability has been guaranteed with 82° phase margin and 88 dB minimum DC-gain. A figure of merit (FOM) must be agreed upon for comparison with previous research works.

The following FOM was defined to highlight the performances of this design with recently published works [53,54,55,56]. This parameter can be explained as the speed-sensitivity product to the power dissipation for a given sensor capacitance. The higher the FOM, the lower the white noise at lower power dissipation [55].

(19) $$FOM=\;\frac{f_{t}}{P_{d}}\;(\mathrm{MHz}/µW)$$

where *P_d_* is the power dissipation and $f_{t}$ being the preamplifier transition frequency. From Table 3, the proposed front-end electronics exhibited a quite high and acceptable FOM of 116.82 MHz/µW. The circuits presented in refs [17,26,49] exhibit higher conversion-gain than that of our design, but they suffer both from higher ENC and low input dynamic. The circuit in ref. [22] has a higher input dynamic of 450 pC and consumes only 2.1 µW of power, but suffers from a very low conversion factor of only 0.044 mV/MeV, involving poor FOM of only 14.29 MHz/µW.

### 3.4. Process Variations

Process variations outcomes worsen with reducing the channel length [52,53]. Mismatch being a function of threshold voltage (VTH) and supply voltage (VDD), low VTH (LVT) transistors have a reduced mismatch impact due to higher VDD/VTH ratio than standard VTH (SVT) or high VTH (HVT) transistors; the proportionate change in temperature from SVT to HVT is much larger as compared to that from LVT to SVT [54,56,57]. Thus, it is more advantageous to move from HVT transistors to SVT devices, but this results in high power dissipation. Large MOS devices increase the intrinsic parasitic capacitances, which leads to more thermal noise, but also reduces local head transfer and mismatch for LVT that can increase the power consumed by the design [52,53,55]. In order to reduce the influence of the high threshold voltage, the input transistors of both the CSA and the pulse shaper modules have been optimized based on conventional LVT operations [57]. In fact, LVT devices have a higher current density and transconductance than regular threshold voltage (RVT) transistors for the same bias conditions, which enforces the previous suitable applications, commented [58,59]. Furthermore, LVT transistors have higher transconductance efficiency, so for low power applications, LVT MOSFETS are recommended. RVT devices have lower VDSsat than LVT MOSFETs. The fact that for applications that need lower supply voltages and do not need require high gains RVT devices are a good choice [58,59]. In addition, LVT transistors present slightly lower parasitic capacitances than RVT transistors, which involves that LVT devices are more suitable for high-frequency applications than the RVT [58,59,60]. Taking into account the trade-off between transistor size and mismatch, we perform optimal transistor sizing/matching with a parallel arrangement of the devices to reduce the parasitic and mismatch effects, canceling, therefore, the short circuit power generated by those parasitic [53,55,60] and achieved 8.72 µW of maximum power consumption. 

## 4. Conclusions

Design techniques of a low-noise, stable and ultra-low power FEE for silicon sensors applications have been described in this research. The design consisted of a compact CSA module linked to a one-order fast PS. The proposed structure was described and analyzed to handle the optimal design parameters. The Spice simulations were therefore implemented and validated by post-layout simulations and Monte Carlo results in 0.35 µm CMOS process, and the specification parameters confirmed the theoretical model. As per FEE design requirements, the input stage transistor aspect ratio has been optimized to guarantee the possible low noise performance. An adjusting gain stage was implemented in the preamplifier stage to control the loop gain and compensated, therefore, the bandwidth limitation of the core amplifier. The feedback resistors were implemented using an active MOS device based voltage-controlled resistor; this allows canceling the parallel noise contribution in the CSA, reducing the energy loss in the shaper feedback capacitance and achieving an amplitude resolution of 1.87% FWHM therefore. The CSA and shaping module achieved a charge to a voltage conversion factor of 546.56 mV/MeV and 920.66 mV/MeV, respectively, verified by the Monte Carlo simulation results, and it is therefore compatible with the state-of-the-art. With a supply voltage of 3.3 V, the readout circuit consumes a maximum power of 8.72 µW and occupied a very low die area of 0.0205 mm^2^. The theoretical analyses together with the post-layout simulations allowed us to prove the functionalities and performance metric of the proposed front-end for ultra-low power and low-noise ROIC for pixel-strip sensors.

## Figures and Tables

**Table 1:** Required CSA specifications for silicon sensors for two vendors [6].

Vendor Parameters	Hamamatsu (H4083)	AMPTEK (A250)
Power consumption	50 mW@12 V	14 m W@6 V
Count rate	2.6 MHz	2.5 MHz
Sensor capacitance	0–25 pF	0–250 pF
ENC (Cin = 5 pF)	240 e^−^	6 e^−^
Noise slope	4 e^−^/pF	11.5 e^−^/pF
Sensitivity	22 mV/MeV (Si)	176 mV/MeV (Si)
DC gain	94 dB	76 dB

**Table 2:** Design parameters of the proposed Front-End Electronics.

Transistor W/L(µm/µm)	gmk Value (µS)	Capacitance/*λ*
M1—62.5/10.5	gm1—61.4	C_L_ = 1 pF
M2—0.84/0.35 M3—18/0.35 M4,5,6—12/0.35	gm2—12.28 gm3—50 gm4,5,6—20	C_m_ = 50 fF C_F_ = 100 fF C_1_ = 0.74 fF
M7—9/0.35	gm7—200	C_2_ = 1.82 fF
M8,9—10/0.35 M_F_—3/36 M_P_—2.772/0.7 M_d_—2/23.6	gm8,9—12 gm_F_—13.13 gm_F_—704.2 gm_d_—2.88	C_d_ = 534 fF C_i_ = 200 fF C_L2_ = 1 pF
M_i_—10/41	gm_i_—3.27	*λ* = 0.0746
M_1sh_—3/20 M_2sh_—0.63/59.25	gm_ish_—4.67 gm_2sh_—0.467 g_03,08_—0.1865	

**Table 3:** Performance comparison of the proposed Front-End Electronics.

Parameters	This Work	[26]	[49]	[45]	[17]	[22]
CMOS Technology	0.35 µm	0.18 µm	0.35 µm	0.13 µm	0.35 µm	0.18 µm
Power Supply	3.3 V	1.8 V	±1.65 V	1.2 V	3.3 V	1.8 V
Power Consumption	8.72 µW	8.7 mW	2.1 mW	4.8 mW	--	2.1 µW
Input Parasitic Capacitance	0.2–2 pF	0.1 pF	17 pF	5 pF	10 pF	-
Gain/Operating Bandwidth	88 dB/1 GHz	--/9.1 GHz	---	-	60 dB/5.1 kHz	-
ENC	37.6 e^−^ + 16.32 e^−^ /pF	278.2 e^−^ + 26.6 e^−^/pF	58.4 e^−^ + 12.7 e^−^/pF	600 e^−^ + 100 e^−^/pF	650 e^−^	-
Amplifier Gain (mV/MeV)	546.56/920.66	513.67/1740.2	9366.45	550.96	826.45	0.044
Active area (mm^2^)	0.0205	0.093	47.64	0.7225	0.75	0.038
Input Dynamic Peaking time	0–280 fC 214 ns	0–15 fC 40 ns/250 ns	6 fC 500 ns/2 us	0–60 fC 100 ns	80 fC ---	450 pC ---
Figure of merit (FOM) (MHz/µW)	116.82	1.05	--	0.002	-	14.29
